# Using Interactive Text Messaging to Improve Diet Quality and Increase Redemption of Foods Approved by the Special Supplemental Nutrition Program for Women, Infants, and Children: Protocol for a Cohort Feasibility Study

**DOI:** 10.2196/32441

**Published:** 2021-12-15

**Authors:** Melissa C Kay, Nour M Hammad, Sharon J Herring, Gary G Bennett

**Affiliations:** 1 Duke University Durham, NC United States; 2 Temple University Philadelphia, PA United States

**Keywords:** WIC, diet quality, digital health, text messaging, mothers, postpartum, child obesity, mobile phone

## Abstract

**Background:**

Children in the United States eat too few fruits, vegetables, and whole grains and too many energy-dense foods; these dietary behaviors are associated with increased risk of obesity. Maternal diet plays a key role in shaping children's diets; however, many mothers have poor diet quality, especially those living in low-income households. The Special Supplemental Nutrition Program for Women, Infants, and Children (WIC) is a federal nutrition assistance program that provides mothers and children with nutrient-dense foods, and those who participate have better diet quality. However, many mothers do not redeem all their WIC-approved foods. Thus, there is a need to create effective interventions to improve diet quality, especially among low-income children and families.

**Objective:**

This paper aims to describe the development and protocol for a study to evaluate the feasibility, satisfaction, and preliminary efficacy of a fully automated text messaging intervention as a strategy to improve maternal diet quality and the redemption of WIC-approved foods.

**Methods:**

We describe the use of the framework developed for the description of nonrandomized feasibility studies. Using an observational, prospective cohort study design, we will recruit mothers enrolled in WIC with a child aged ≤2 years. Participants will receive automated SMS text messages aimed at improving the redemption of WIC-approved foods to improve the participants’ diet quality for 12 weeks. All outcome measures will be analyzed using descriptive and inferential statistics. Qualitative data will be analyzed using thematic analysis.

**Results:**

Data collection for this study began in March 2021. We expect the study results to be available within 9 months of study commencement. The results will shed light on the feasibility, acceptability, and effectiveness of using automated text messages as a behavior change strategy for mothers enrolled in WIC.

**Conclusions:**

The results of this pilot study will explore whether this digital behavioral intervention, which will deliver nutrition guidance in accordance with the Dietary Guidelines for Americans using interactive self-monitoring and feedback, is feasible and acceptable. This will lay the foundation for a larger evaluation to determine efficacy for improving diet quality in those most at risk for obesity.

**Trial Registration:**

ClinicalTrials.gov NCT04098016; https://clinicaltrials.gov/ct2/show/NCT04098016

**International Registered Report Identifier (IRRID):**

DERR1-10.2196/32441

## Introduction

### Background

The etiology of childhood obesity is multifaceted and is largely influenced by diet quality and the consumption of obesogenic foods [1,2]. Maternal diet is a critical driver of the child’s diet and can be a risk factor for childhood obesity [3-5]. Mothers shape the food environment by choosing which foods to buy and demonstrating what foods to eat through their own observed eating behaviors [6]. However, for many mothers, especially those living in low-income households, diet quality is suboptimal [3]. As such, many children consume sugar-sweetened beverages, desserts, and sweets from as early as the age of 4 months [7-11]. Thus, intervening on maternal diet quality during infancy may benefit the eating patterns of both the mother and child and reduce their subsequent obesity risk [3]. However, few interventions focus on improving diet quality for mothers, particularly during the postpartum period.

The Special Supplemental Nutrition Program for Women, Infants, and Children (WIC) is a federal nutrition assistance program that supports low-income, nutritionally at-risk pregnant and postpartum women, along with infants and children up to the age of 5 years. In 2020, WIC services were provided to nearly 6.3 million women and young children in the United States [12]. Participants in WIC receive nutrition education, breastfeeding support, referrals to other health and social services, and monthly benefits for specific foods (eg, milk, eggs, bread, and cereal) in specific quantities [13]. WIC also provides a small cash amount for fruits and vegetables *only* (cash value vouchers). Participation in the WIC program contributes to improved diet quality for both the mother and child [14,15], a reduction in the incidence of overweight and obesity [16-18], and cost savings in health care [19].

However, WIC is experiencing a steady decline in caseloads [20]. This may be due to required visits with WIC nutritionists to maintain the recipients’ eligibility to continue receiving benefits. At these visits, women have their anthropometrics measured, are educated about infant feeding, and set goals for improving nutrition for themselves and their children. However, there is little follow-up with goal setting between visits, and many WIC beneficiaries report dissatisfaction with the nutrition education sessions, citing them as repetitive, long, or not very useful [21]. In addition, many mothers do not fully redeem their food benefits (ie, purchasing all items in the full quantities assigned), missing out on some of the nutritional benefits the program is designed to provide [22,23]. Efforts are needed to support these required nutrition education sessions and aid in the maintenance of goals, reinforce progress, and problem solve barriers to achieving goals.

Digital health interventions may serve as a scalable, cost-effective, and efficacious way to support mothers enrolled in WIC [24,25]. Nearly all adults (99%) in the United States have a cell phone, and 85% own smartphones; many Americans with lower incomes and those of childbearing age (18-29 years) rely only on smartphones for internet access [26,27]. Many programs have shown promise in successfully using digital technologies among parents to address health issues, including vegetable intake, sedentary behavior, bedtime routines, breastfeeding, and oral health [28-32]. Given their low cost, reach, and dissemination potential, using cell phones to deliver behavioral interventions could bridge gaps in health disparities and enable access to information across sociodemographic groups [25].

Feasibility studies are essential for exploring the relevance and acceptability of interventions [33]. Findings from feasibility studies can help determine whether an intervention should be tested for efficacy. The use of rigorous standards to guide the design and evaluation of feasibility studies is essential, though not often practiced. Here, we describe the use of a framework described by Bowen et al [34], which provides guidance for conducting nonrandomized feasibility studies. Indications for this feasibility trial include a lack of published studies focused on improving the redemption of WIC-approved foods and uncovering the reasons for beneficiaries not redeeming all their WIC benefits.

This paper describes the development and implementation and evaluation plans for *Healthy Roots, *a fully automated, pilot 12-week prospective cohort study of a digital behavioral intervention aimed at improving the redemption of WIC-approved foods to improve diet quality.

### Study Aims and Objectives

The aims of this study are as follows: (1) to determine the feasibility of the intervention and (2) to determine the preliminary efficacy of the intervention on changes in maternal diet quality and the redemption of WIC-approved foods. Thus, the primary objectives of this paper are to describe the rationale and design of Healthy Roots using a rigorous framework to enable the reporting of a detailed assessment of the feasibility findings. These findings will guide intervention refinements and inform the appropriateness of a future randomized controlled efficacy trial.

The study has been approved by the Duke Health Institutional Review Board and is registered with ClinicalTrials.gov (NCT04098016). Electronic written informed consent will be obtained from the study participants.

## Methods

### Overview

The intervention was designed in 2 parts according to questions outlined by Bowen et al [34]. The initial phase focused on answering the question, *Can it work?* We engaged both WIC nutritionists and beneficiaries to conceptualize and design the intervention to answer this question. We used a qualitative approach centered on 2 areas of focus: acceptability and demand, as described in the following sections.

### Part 1: Formative Work

#### Acceptability and Demand

According to Bowen et al [34], assessing acceptability during the intervention design phase allows researchers to understand how the intervention will fit into participants’ daily lives. Assessing demand allows us to determine whether WIC beneficiaries will use the intervention to guide their behaviors and choices. Therefore, before conducting the pilot, we conducted in-depth semistructured interviews with parents or legal guardians and caregivers (hereafter referred to as parents) enrolled in WIC (N=13) via telephone. To be considered eligible, the parents must be receiving assistance from WIC, have a child aged ≤2 years, have a cell phone that can receive SMS text messages, and be English or Spanish speakers. To assess the acceptability, parents were asked to list the WIC-approved foods they were most likely to redeem and the foods they were least likely to redeem as well as the reasons affecting their food choices. They were also asked to describe the recipes and cooking methods used to prepare those foods. Parents were asked to specify their thoughts and practices about healthy eating, and how WIC does or does not help them eat healthily. To assess demand, they were also asked questions regarding their digital preferences, such as the preferred frequency of receiving text messages and the desired content of the text messages.

#### Analysis and Results

We used the Rapid Identification of Themes from Audio Recordings (RITA) procedure to conduct a rapid assessment of the interviews [35]. RITA allows for the expeditious identification of themes without the time-consuming and costly process of transcription, while minimizing the loss of information that often accompanies alternative rapid analysis procedures [36]. We used results from RITA to guide intervention development. We identified the following foods as the most commonly purchased food items: fruits and vegetables, milk, cheese, eggs, and cereal. The reasons behind these purchases include taste, child preferences, health benefits, and use in meal preparation. The least commonly purchased food items were peanut butter and yogurt. Parents typically did not purchase these foods because of child taste preferences and because they come in large packages, increasing their likelihood of going to waste. The parents used various sources to determine how to use their WIC-approved foods, including phone apps, food shows, internet recipes, cookbooks, and family recipes. Most reported cooking and eating at home at least four to five times per week. When asked about healthy eating, most parents noted the importance of consuming high amounts of fruits and vegetables and smaller portion sizes, whereas unhealthy eating was associated with the consumption of processed foods high in sodium or sugar or both. All parents believed that a text messaging program would be helpful in improving their diet quality. The reasons included wanting assistance with eating healthy and using the foods they received in their WIC packages. Parents reported that text messages are an optimal way to reach parents because “nearly everyone has a smartphone” and people send text messages more frequently than emails. As for the content of the text messages, most parents wanted to receive recipes and information about nutritional value, portion control, and meal preparation. The preferred frequency for the text messages varied by about half, with the participants preferring to receive text messages weekly and in the morning.

### Part 2: Intervention Development and Implementation

#### Overview

The second part of the intervention design focuses on answering the question, *Does it work?* [34]. We used results from the formative work to guide the development of the content, frequency, and timing of text messages to ensure that the program matched the preferences and needs identified by the WIC beneficiaries. We used parents’ feedback to tailor the goals, tips, and recipes to meet the needs of WIC beneficiaries while aiming to increase their diet quality by increasing the consumption of WIC-approved foods. The intervention is delivered electronically and is fully automated. It was adapted from an evidence-based digital obesity treatment program called the interactive obesity treatment approach (iOTA) [37-43]. iOTA was developed by GGB and the Duke Global Digital Health Science Center and has been tested in previous trials, including adaptation for other populations such as parents of children with obesity [44]. Similar to iOTA, intervention components in this study will be delivered using SMS text messages, interconnected algorithms, and content libraries [45]. Intervention development was also informed by the Social Cognitive Theory to include effective behavior change techniques, such as self-monitoring and goal setting [46,47].

#### Behavioral Change Goals

Goal creation was guided by formative work and focused on foods identified as being the least likely to be redeemed. In addition, goals were based on their empirical support for improving diet quality, ease of self-monitoring, and concreteness. Goals and tips were developed specifically for WIC beneficiaries using resources recommended by WIC, including guidelines on developing digital tools for WIC participants. WIC nutritionists aided in the development of goals and provided feedback on the message content. Messages were refined based on feedback, and user testing was conducted (N=8); the messages were updated accordingly and finalized by the team’s registered dietitians (MCK and NMH).

Each of the 6 goals focuses on a specific set of WIC-approved foods as identified in our formative work. They include nutrient-dense foods such as fruits, vegetables, beans, whole grains, nuts, peanut butter, and leafy green vegetables. See Table 1 for an overview of the goals, the goal tracking questions (ie, self-monitoring), and the optimal answers to the tracking questions. For each goal, the recommended amount and frequency of consumption was guided by the 2020-2025 Dietary Guidelines for Americans [48]. Tips include recipes and simple behaviors to support meeting the goal. All recipe links are from organizations that serve WIC beneficiaries.

**Table 1:** Healthy Roots behavior change goals and tracking questions.

Ways to increase diet quality	WIC^a^-approved foods	Goal	Tracking question	Optimal answer
Increase fruits	Fruit	For the next 2 weeks aim to eat 2 fruits or more each day	Over the past week, on how many days did you eat 2 fruits or more?	≥2
Increase vegetables	Vegetables	For the next 2 weeks try to eat 3 vegetables or more each day	Over the past week, on how many days did you eat 3 vegetables or more?	≥3
Increase greens and beans	Legumes	Your goal for the next 2 weeks is to eat beans 2 times or more each week	Over the past week, how many times did you eat beans (like kidney, navy, or pinto beans; chickpeas; or lentils)?	≥2
Increase whole grains	Bread, tortillas, pasta, and cereal	For the next 2 weeks eat 3 or more whole grains each day	Over the past week, on how many days did you eat 3 or more whole grains?	≥3
Increase plant proteins	Legumes	For the next 2 weeks eat nuts or peanut butter 3 times or more each week	Over the past week, how many times did you eat nuts or peanut butter?	≥3
Increase dark green vegetables	Vegetables	For the next 2 weeks your last goal is to eat leafy green vegetables (like spinach, lettuce, bok choy, Swiss chard, collards, or kale) 2 times or more each week	Over the past week, how many did you have leafy green vegetables?	≥2

^a^WIC: Special Supplemental Nutrition Program for Women, Infants, and Children.

#### Self-monitoring With Tailored Feedback Messages

Regular self-monitoring is an important predictor of behavior change [49]. To enhance engagement with the intervention and foster behavior change, participants will be asked to self-monitor their behavior weekly via SMS text messaging throughout the intervention period. Participants will receive a text message prompting them to communicate their weekly tracking data; for example,

Time to check in. Over the past week, on how many DAYS did you eat 2 fruits or more? Please text only a number (like 1, 2, 5). Do not text words.

**Figure 1:**
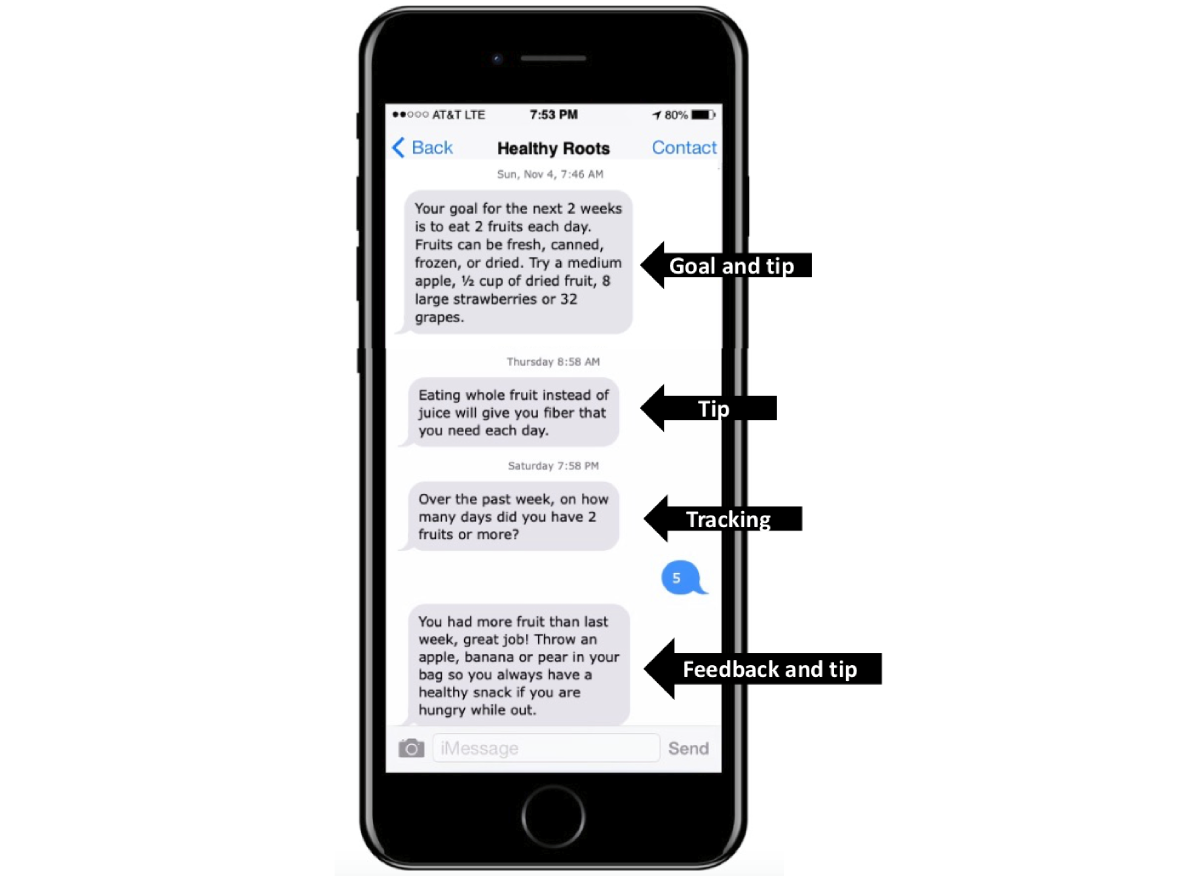
SMS text message exchange showing tips, tracking, and feedback.

The computer algorithm will then determine which feedback message to send according to the participant’s response. Participants who provide self-monitoring data via SMS text messages will immediately receive tailored feedback and a brief skills training message (Figure 1). Feedback messages will describe progress, reinforce successes, offer motivational strategies, and provide short skills training tips. A retry protocol will attempt to reach the participants if the first SMS text message goes unanswered.

### Population

#### Demographics

We will enroll mothers aged ≥18 years who have a child aged ≤2 years and who are currently enrolled in WIC in North Carolina, United States. Parents must have a smartphone (ie, a cell phone that can send and receive text messages and access the internet), be willing to send and receive study-related text messages, have an email address they check regularly, have regular access to the internet, and be comfortable reading and writing in English.

#### Sample Size

Given that this is a feasibility study, we did not use traditional sample size calculations because hypothesis testing is an aim of this study [50,51]. When determining sample size, we consulted the recommendations for good practice in the design of pilot and feasibility studies, where recommendations vary from 12 to 50 participants [52-55]. Thus, we based our sample size on the following objectives: (1) test the integrity of the study protocol, (2) estimate rates of recruitment and consent, and (3) determine the acceptability of the intervention. In addition, because we are also interested in estimating the intake of episodically consumed foods, such as vegetables and whole grains, we chose a slightly larger sample size. For this study, we will recruit a total of 50 parents enrolled in WIC.

#### Recruitment

We have partnered with 2 different entities for participant recruitment using different strategies. The first is a private, nonprofit health system that operates federally qualified community health centers in North Carolina, United States, which also administer WIC benefits. During WIC encounters, the WIC nutritionists (n=2) will upload the name, phone number, and email address of WIC beneficiaries who are interested in joining the pilot to a secure web-based Duke Box folder. Research assistants will upload this information into REDCap (Research Electronic Data Capture), a secure, web-based software platform designed to support data capture and management for research studies [56,57]. The second recruitment entity is the WIC office from the second most populated county in North Carolina. This county WIC office will include flyers in mailings sent to all new WIC recipients. These flyers will provide information about the pilot study and will include a QR code that parents can scan to take them directly to the screener.

In addition, the Nutrition Services Branch within the North Carolina Division of Public Health, which implements the WIC program for North Carolina, United States, will alert all WIC directors in the state of the pilot study via email. The email will include the flyer with the QR code for WIC directors to distribute throughout their WIC clinics as they see fit. We will also use social media platforms, such as Facebook (Facebook Inc) and Duke University websites, to distribute information about the study and aid in recruitment. Interested parents can also find a link to the screener from a study-specific website. This website contains the study and contact information for the research team.

#### Screening, Baseline Assessments, and Enrollment

All study-related processes will be completed electronically to aid in the dissemination potential. The surveys will be administered through REDCap, allowing parents to self-guide themselves through each one. The system incorporates functions such as scheduling surveys and tracking their completion. If surveys are not complete, the system will automatically send reminders via SMS text messages and email, for up to 7 days.

**Figure 2:**
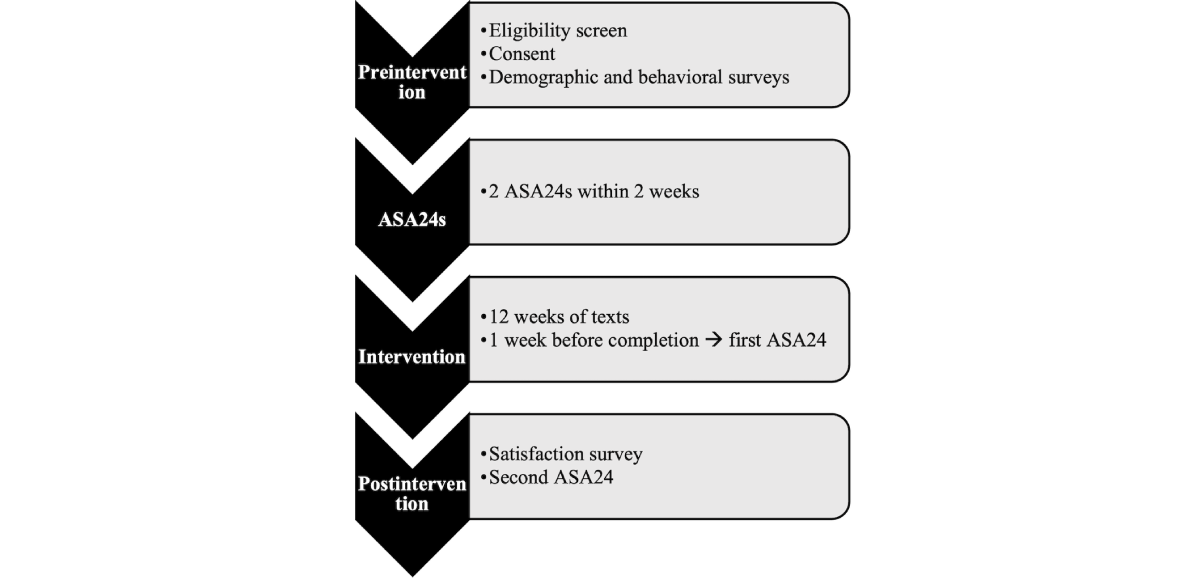
Healthy Roots study flow. ASA24: Automated Self-Administered 24-Hour Dietary Assessment Tool.

The first survey is the eligibility screener, which includes questions regarding eligibility criteria. As soon as contact information is entered into REDCap by the research assistant, the system will automatically send an email with a link to the web-based screener and will send a SMS text message the following day if not complete. Interested parents will also be able to access the screener by scanning the QR code on the flyer or clicking on the screener link found on the study website and Facebook page. If a parent is eligible, they will confirm their contact information and be directed to a web-based consent form (see Figure 2 for study flow). Once the consent form is electronically signed, the parents will be directed to complete a web-based baseline survey.

Upon completion of the baseline survey, participants will be asked via email to complete two 24-hour dietary recall assessments administered through the Automated Self-Administered 24-Hour Dietary Assessment Tool (ASA24) [58]. The ASA24 is a freely available, web-based, self-administered tool developed by the National Cancer Institute and provides comprehensive nutrient data on all foods and beverages consumed during the previous 24-hour period. Parents will be asked to complete 2 separate 24-hour dietary recalls (1 during the week and 1 in the weekend) within 2 weeks of each other. When both ASA24s are complete, parents will be enrolled in the intervention.

Once the participants are enrolled, they will begin receiving SMS text messages. The first text message will include a web link to a study-specific YouTube (Google Inc) channel that includes an orientation video. This 2-minute orientation video informs the participants of what to expect, including surveys they have to complete and the incentives for completing those surveys. They are presented with the study schedule and contact information for the study team and encouraged to reach out with any questions or concerns. Subsequently, participants will receive their first goal, followed by a new goal every 2 weeks until the end of the study for a total of 6 goals. During each 2-week goal cycle, participants will receive 3 tips per week related to that goal. At the end of each week, they will receive a tracking message related to their assigned goal. If they respond, they will receive feedback on their progress. If they do not respond, they will receive an automated reminder. If there is still no response, they will receive a reminder to track the next time and a tip. After 12 weeks, participants will be asked to complete 2 more ASA24s and a satisfaction survey. The participants will receive a gift card after the completion of each ASA24 (n=4) for a maximum amount of US $55.

### Data Collection and Outcome Assessments

Self-reported sociodemographic characteristics, including education, age, BMI (kg/m^2^), marital status, employment, depression, race, and ethnicity will be collected before enrollment through REDCap web-based surveys (Table 2). REDCap will also be used to capture intervention retention defined as responding to weekly tracking surveys. REDCap will also be used to capture postintervention satisfaction surveys. Research staff will follow up with phone calls and text messages for those who do not complete the satisfaction survey.

**Table 2:** Schedule of assessments.

Measure	Screening	Baseline	12 weeks
Inclusion and exclusion criteria	✓		
Informed consent	✓		
Sociodemographics		✓	
Food insecurity^a^		✓	
WIC^b^ shopping habits		✓	✓
Depression^c^		✓	
Everyday discrimination^d^		✓	
ASA24^e^ dietary tracking		✓	✓
Satisfaction survey			✓

^a^2-item screen for food insecurity [59].

^b^WIC: Special Supplemental Nutrition Program for Women, Infants, and Children.

^c^Using personal health questionnaire depression scale (PHQ-8) [60].

^d^Using expanded everyday discrimination scale [61].

^e^ASA24: Automated Self-Administered 24-Hour Dietary Assessment Tool.

### Primary Outcomes

Feasibility will be assessed using a mixed methods approach across 5 domains based on the framework described by Bowen et al [34] to answer the question *Does it work?* Table 3 provides an overview of each domain and its outcome.

**Table 3:** Description of feasibility domains for assessment.

Domain	Description	Data source and measure
Acceptability	The extent to which the intervention (delivery and content) is considered relevant, appropriate, or satisfying to program participants and WIC^a^ nutritionists	Quantitative process evaluation and program satisfaction survey with participants Qualitative process evaluation with participants (n=12) Quantitative process evaluation with WIC nutritionists (n=4)
Demand or reach	The extent to which the intervention is likely to be used and the reach among the intended population	Total number of WIC beneficiaries reached to recruit sample size Time to recruit required sample size Number of eligible participants needed to recruit required sample size Number of participants who enrolled and completed the study
Implementation	The extent to which the intervention can be fully implemented as planned	Number of participants who complete all aspects of the intervention Number of SMS text messages successfully delivered Number of participants who respond to each of the SMS tracking messages
Preliminary efficacy	The extent to which the intervention works in making positive changes to diet quality and improved redemption of WIC-approved foods	Estimated changes in diet quality as measured by the Healthy Eating Index-2015 using ASA24^b^ dietary recall data [62] Changes in self-reported purchasing of WIC-approved foods

^a^WIC: Special Supplemental Nutrition Program for Women, Infants, and Children.

^b^ASA24: Automated Self-Administered 24-Hour Dietary Assessment Tool.

*Acceptability* of the intervention and study procedures will be assessed both quantitatively and qualitatively. Quantitative data will be collected via a poststudy satisfaction survey to assess the acceptability of the message frequency, their timing and content, and perceptions about the use of digital technologies for improving diet quality. We will also survey WIC nutritionists (n=4) to gather feedback on recruitment and thoughts on the program. An embedded qualitative study will obtain the participants’ views and experiences of the intervention. In-depth interviews will be conducted on a sample of at least 12 participants to allow for data saturation when assessing barriers and facilitators to healthy eating [63]. We will use a purposive sampling strategy to ensure that we obtain feedback from both high and low engagers with the intervention to capture all perspectives on participating in the study [64]. Interview guides will be developed to ensure consistency across interviews but also to allow for probing, as deemed appropriate. All interviews will be audio-recorded with responses kept confidential. The participants will receive a US $15 gift card as an incentive.

*Reach and demand* for the intervention will be measured using administrative and survey data, including participant enrollment, sociodemographics, and retention at the 12-week survey.

*Implementation* will be assessed through intervention engagement, calculated by dividing the number of days with valid tracking data (numerator) by the total number of possible tracking days (denominator). We will calculate and compare engagement rates by the week. We will create a dichotomous outcome variable comparing high and low engagers using an established cutoff of 80% or more engagement in weekly self-monitoring [65,66]. Bivariate analyses using 2-tailed *t* tests and chi-square tests will be used to examine predictors of intervention engagement. Poisson regression with a robust variance will be used to examine sociodemographic differences among those with higher levels of engagement (80% or more weeks of tracking) and estimate risk ratios and 95% CIs.

*Preliminary efficacy* will be measured by assessing changes in diet quality and self-reported WIC-approved food preferences. Participants will be asked about their purchasing habits before and after the intervention and the reasons for their choices. The embedded qualitative study will also obtain a more in-depth assessment of changes in WIC food purchases in a subset of the participants. Dietary intake will be measured using the ASA24 recall tool. Participants will be asked via email to complete 2 separate dietary recalls (1 during the week and 1 in the weekend) within a 14-day period before enrollment and again upon study completion, for a total of 4 dietary recalls. These data will be used to calculate a Healthy Eating Index (HEI)-2015 score, which consists of 13 components, 9 of which assess adequacy of the diet, including: (1) total fruit, (2) whole fruit, (3) total vegetables, (4) greens and beans (including peas), (5) whole grains, (6) dairy, (7) total protein foods, (8) seafood and plant proteins, and (9) fatty acids, which is a ratio of poly- and mono-unsaturated to saturated fatty acids. The remaining 4 assess dietary components recommended to be consumed in moderation: (10) refined grains, (11) sodium, (12) added sugars, and (13) saturated fats [62]. For all components, higher scores reflect better diet quality as moderation components are reverse scored. Each component is scored on a density basis rather than absolute scores, either as a percentage of calories or per 1000 calories, allowing use of the HEI for a range of ages and populations. Summed scores of the 13 components yield a maximum total score of 100, with a higher score reflecting greater compliance with the 2015-2020 Dietary Guidelines for Americans [67].

### Analytic Approach

#### Quantitative Data Analysis

Data from this pilot study will be descriptive with outcomes being estimates of variables related to feasibility [34]. Sample characteristics will be described as frequencies for categorical variables and as means for continuous variables. HEI-2015 scores will be summarized as means, SDs, and the percent maximum score ([mean score/maximum score] × 100%]. We will explore intervention effects on changes in HEI score from baseline to 12 weeks using a 2-sample *t* test. We will conduct exploratory analyses to assess differences in HEI scores between low and high engagers using linear regression. A similar analysis will be conducted for changes in the relevant behavioral and psychosocial variables. If the distribution of any outcome is heavily skewed, we will either appropriately transform the data so that it is normally distributed or use a generalized linear mixed model with an appropriate link function. Sensitivity analyses will fit linear mixed models with a full maximum likelihood estimation using all available data to allow for responses to be missing at random, where the missing mechanism may be related to either observed covariates or response variables but not related to the unobserved data. All analyses will be conducted using STATA 14 (StataCorp, Inc).

#### Qualitative Data Analysis

All interviews with the participants and WIC nutritionists will be audiotaped and transcribed verbatim. In contrast to part 1, which used the RITA method, a 2-stage process will be followed to analyze interview transcripts on an iterative basis for refinement of interview guides and auditing of transcript quality. The first interview transcript will be pilot coded independently by 2 researchers to agree on a coding strategy (ie, to ensure that both researchers are coding consistently and to discuss and resolve any differences). The initial findings of the transcript will be discussed before coding the remaining transcripts. Data from the remaining transcripts will be independently coded by the 2 researchers. This will involve reading and rereading transcripts, identifying themes and subthemes, and mapping these with supporting salient quotes to an appropriate theoretical domain. Content analysis will identify patterns, make subgroup comparisons, and identify relationships within and between major themes using NVivo 12 (QSR International), which is the qualitative data analysis software developed to manage the *coding* procedure [67].

## Results

Data collection for this study began in March 2021. We expect the study results to be available within 9 months of the study commencement. The findings will shed light on the feasibility, acceptability, and effectiveness of using automated text messages as a behavior change strategy for mothers enrolled in WIC. The study was approved by the Duke Health Institutional Review Board and registered with ClinicalTrials.gov NCT04098016. Electronic written informed consent was obtained from the study participants.

## Discussion

### Overview

The postpartum period is an opportune time to provide nutrition support to women as several studies indicate a decrease in diet quality and increased risk for obesity [68-70]. In addition, intervening during the postpartum period has benefits for the child as well; children’s development of food preferences and eating habits are heavily influenced by their mothers during this time [71]. However, few studies have explored intervention effects on maternal diet in the postpartum period, particularly among those most vulnerable [72]. Our study offers an opportunity to not only improve retention in WIC and redemption of WIC-approved foods but also improve diet quality, something few studies aim to do [24].

Mothers participating in WIC are a priority population as it is the largest program providing services to improve the nutritional status of women and young children in the United States [73]. Although many women receive nutrition support through WIC, there are many barriers to its uptake, and women may prefer using digital methods to receive nutrition education. Digital health interventions are a scalable and efficacious way to support women enrolled in WIC because mobile phone use is ubiquitous across racial, ethnic, and socioeconomic groups in the United States [27]. A well-designed digital intervention that leverages the reach of WIC has the potential to be cost-effective and scalable to accommodate the disparities in obesity risk among high-risk groups.

Text messaging interventions can be cost-effective as they can reach large groups of people at a low cost per person compared with more complex interventions [74]. The pre- to postnatal period is an opportune time to use text messaging as mothers have a strong need and desire to obtain pregnancy- and child health–related information [75-77]. This is evidenced by the over 1 million subscribers to Text4Baby, a text messaging program that delivers health and safety information about pregnancy and the first year of infancy [78]. Text messaging is an enticing intervention strategy as messages can be stored, read, and answered at the participant’s convenience; they are relatively inexpensive, are available for any type of phone, and have had a positive impact on many behavioral outcomes [74,79].

This study uses a rigorous feasibility assessment framework [34] to provide preliminary evidence on how digital technologies can impact engagement and retention in WIC and insight into the adoption potential of digital interventions within the WIC workflow. Seeking input from WIC participants directly, as they are the end users, provides valuable input regarding the development of text message content and support acceptability and engagement with the intervention. Using a framework for investigating feasibility will allow us to further understand the strengths and weaknesses of the intervention idea, that is, acceptability (beneficiaries and nutritionists), demand, implementation, and practicality, along with limited efficacy.

### Limitations

A limitation of this pilot feasibility study is the 1-group design and small sample size, which prevents us from making definitive statements regarding its effectiveness. Limitations can be mitigated by the mixed methods approach to the design of the intervention, which will provide useful insights. The study sample might experience selection bias, based on the necessity of having a smartphone and an email address to be able to participate, which may limit the generalizability of the findings. However, 85% of adults in the United States own a smartphone, with higher levels of ownership among adults of childbearing age [27]. Moreover, the collection of data using self-reported measures has limitations in addition to the burden of required measures, such as the ASA24. There may be participants who are unable to respond to the measures without assistance. We will mitigate this with a research assistant who will reach out to those with incomplete data to assist in completion; this may lengthen the process of data collection but will ensure the completeness of data across all participants.

### Conclusions

If successful, Healthy Roots will provide evidence to support scalable text message interventions as an appropriate tool to complement existing WIC services. The findings have the potential to inform the National WIC Association on the scalability and translation of the intervention. If the intervention is effective, it could provide benefits at a population-, individual-, and health service delivery–level by improving retention and diet quality with a low-cost, low–resource intensive intervention that can enhance existing models of care. Continued efforts are needed to improve the WIC experience and maximize program efficacy. During the current COVID-19 pandemic, this is particularly important as programs seek alternatives to engage with participants where possible [80]. The results from this pilot study will help explore whether this digital behavioral intervention, which will deliver nutrition guidance for meeting recommendations outlined in the Dietary Guidelines for Americans using interactive self-monitoring and feedback, is feasible and acceptable. This will lay the foundation for a larger evaluation to determine whether this intervention is effective at scale for improving diet quality in those at risk and will help determine the potential for this intervention to be implemented in the existing WIC infrastructure.

## Abbreviations

ASA24: Automated Self-Administered 24-Hour Dietary Assessment Tool
HEI: Healthy Eating Index
iOTA: interactive obesity treatment approach
REDCap: Research Electronic Data Capture
RITA: Rapid Identification of Themes from Audio Recordings
WIC: Special Supplemental Nutrition Program for Women, Infants, and Children
